# Identification of lipid-modifying drug targets for autoimmune diseases: insights from drug target mendelian randomization

**DOI:** 10.1186/s12944-024-02181-2

**Published:** 2024-06-22

**Authors:** Xiao Hu, Peng Zhang, Yuan Gao, Wen-Wen Ding, Xue-Er Cheng, Qian-Qian Shi, Sheng Li, Yan-Yu Zhu, Hai-Feng Pan, Peng Wang

**Affiliations:** 1 Department of Health Promotion and Behavioral Sciences, School of Public Health, Anhui Medical University, 81 Meishan Road, Hefei, Anhui 230032 China; 2 Institute of Kidney Disease, Inflammation & Immunity Mediated Diseases, The Second Hospital of Anhui Medical University, Hefei, China; 3 Department of Epidemiology and Biostatistics, School of Public Health, Anhui Medical University, 81 Meishan Road, Hefei, Anhui 230032 China; 4 Health Services and Management, School of Health Management, Anhui Medical University, 81 Meishan Road, Hefei, Anhui 230032 China; 5 The Second Clinical School of Medicine, Anhui Medical University, 81 Meishan Road, Hefei, Anhui 230032 China

**Keywords:** Lipid-lowering drugs, Autoimmune diseases, Rheumatoid arthritis, Mendelian randomization

## Abstract

**Backgrounds:**

A growing body of evidence has highlighted the interactions of lipids metabolism and immune regulation. Nevertheless, there is still a lack of evidence regarding the causality between lipids and autoimmune diseases (ADs), as well as their possibility as drug targets for ADs.

**Objectives:**

This study was conducted to comprehensively understand the casual associations between lipid traits and ADs, and evaluate the therapeutic possibility of lipid-lowering drug targets on ADs.

**Methods:**

Genetic variants for lipid traits and variants encoding targets of various lipid-lowering drugs were derived from Global Lipid Genetics Consortium (GLGC) and verified in Drug Bank. Summary data of ADs were obtained from MRC Integrative Epidemiology Unit (MER-IEU) database and FinnGen consortium, respectively. The causal inferences between lipid traits/genetic agents of lipid-lowering targets and ADs were evaluated by Mendelian randomization (MR), summary data-based MR (SMR), and multivariable MR (MVMR) analyses. Enrichment analysis and protein interaction network were employed to reveal the functional characteristics and biological relevance of potential therapeutic lipid-lowering targets.

**Results:**

There was no evidence of causal effects regarding 5 lipid traits and 9 lipid-lowering drug targets on ADs. Genetically proxied 3-hydroxy-3-methylglutaryl-CoA reductase (HMGCR) inhibition was associated with a reduced risk of rheumatoid arthritis (RA) in both discovery (OR [odds ratio] = 0.45, 95%CI: 0.32, 0.63, *P* = 6.79 × 10^− 06^) and replicate datasets (OR = 0.37, 95%CI: 0.23, 0.61, *P* = 7.81 × 10^− 05^). SMR analyses supported that genetically proxied HMGCR inhibition had causal effects on RA in whole blood (OR = 0.48, 95%CI: 0.29, 0.82, *P* = 6.86 × 10^− 03^) and skeletal muscle sites (OR = 0.75, 95%CI: 0.56, 0.99, *P* = 4.48 × 10^− 02^). After controlling for blood pressure, body mass index (BMI), smoking and drinking alchohol, HMGCR suppression showed a direct causal effect on a lower risk of RA (OR = 0.33, 95%CI: 0.40, 0.96, *P* = 0.042).

**Conclusions:**

Our study reveals causal links of genetically proxied HMGCR inhibition (lipid-lowering drug targets) and HMGCR expression inhibition with a decreased risk of RA, suggesting that HMGCR may serve as candidate drug targets for the treatment and prevention of RA.

**Supplementary Information:**

The online version contains supplementary material available at 10.1186/s12944-024-02181-2.

## Introduction

Autoimmune diseases (ADs) are chronic inflammatory connective tissue disorders characterized by the loss of immunological tolerance to self-antigens and an overactive immune response against healthy cells or tissues, causing multi-organs damage and the production of autoantibodies [1]. It has been estimated that ADs affect approximately 11% of the population worldwide, and the prevalence will continue to increase over time [2, 3]. During the past two decades, a large number of literature has demonstrated that genetic susceptibility, environmental factors, sex hormones, and immunological regulation dysfunction are generally considered to contribute to the onset and development of ADs [4]. Currently, the available medications for the treatment of ADs only provide symptomatic relief rather than a complete cure, although injectable biologics are well-established targeted therapy for ADs, the high out-of-pocket medication costs are a deterrent to patient adherence [5].

Lipids are fatty compounds that play important roles in regulating energy balance, contributing to cellular structural integrity and function, facilitating hormone production, etc [6, 7]. A growing body of literature has emphasized the pivotal role of lipid metabolism in supporting an effective immune response, modulating inflammatory effects, and facilitating tissue regeneration. Whereas, lipid metabolism dysregulation has been recognized as a critical factor participating in the pathogenesis of cardiovascular diseases (CVD), metabolic diseases, and Inflammatory disorders [8, 9]. In addition to these connections, the emergence of novel lipid-lowering agents, such as proprotein convertase subtilisin/kexin type 9 (PCSK9) and Niemann-Pick C1-like intracellular cholesterol transporter 1 (NPC1L1), has been introduced as potential drug targets for therapeutic development of a variety of diseases. Literature has indicated that these inhibitors are not only effective in lowering lipid levels, but may also have significant relevance in the treatment of heart failure, rheumatoid arthritis (RA), and systemic lupus erythematosus (SLE) [10, 11]. Although several studies have explored the association between lipid-lowering drugs and ADs, the findings have not yielded consistent conclusions [12–14]. Furthermore, it is crucial to acknowledge that previous observational studies may be affected by confounding factors, which can obscure the establishment of causal relationships.

Mendelian randomization (MR) is a novel biostatistical approach that uses genetic variants as instrumental variables (IVs) to infer causal associations between exposure factors (biomarkers or drug targets) and outcomes. As the transmission of genetic variants is naturally inherited from a parent at conception, MR is less affected by confounding factors and reverse causation in contrast to observational epidemiological studies. Given that genetic variants within the genes encoding region targets can affect the expression or functions of target genes, it is analogous to the mechanisms of actions of drugs, therefore, in drug target MR, genetic variants, represent proxies for an intervention on the proposed drug target, are leveraged as IVs to explore potential effects of drug target on disease outcomes [15–18].

In this study, we conducted univariable two-sample MR analyses to initially assess causal associations of lipid traits and lipid-lowering drug targets on the risks of ADs. Then, summary data-based MR (SMR) analyses were implemented to validate the associations of lipid-lowering drug targets and ADs using expression quantitative trait loci (eQTL) data in whole blood and multiple tissues. After adjusting for potential confounding factors by multivariable MR (MVMR), we performed enrich analyses and protein-protein interaction (PPI) networks to understand the biological significance and underlying interactions between lipid-lowering drug targets and the approved therapeutic targets of ADs.

## Materials and methods

### Study design

**Fig. 1:**
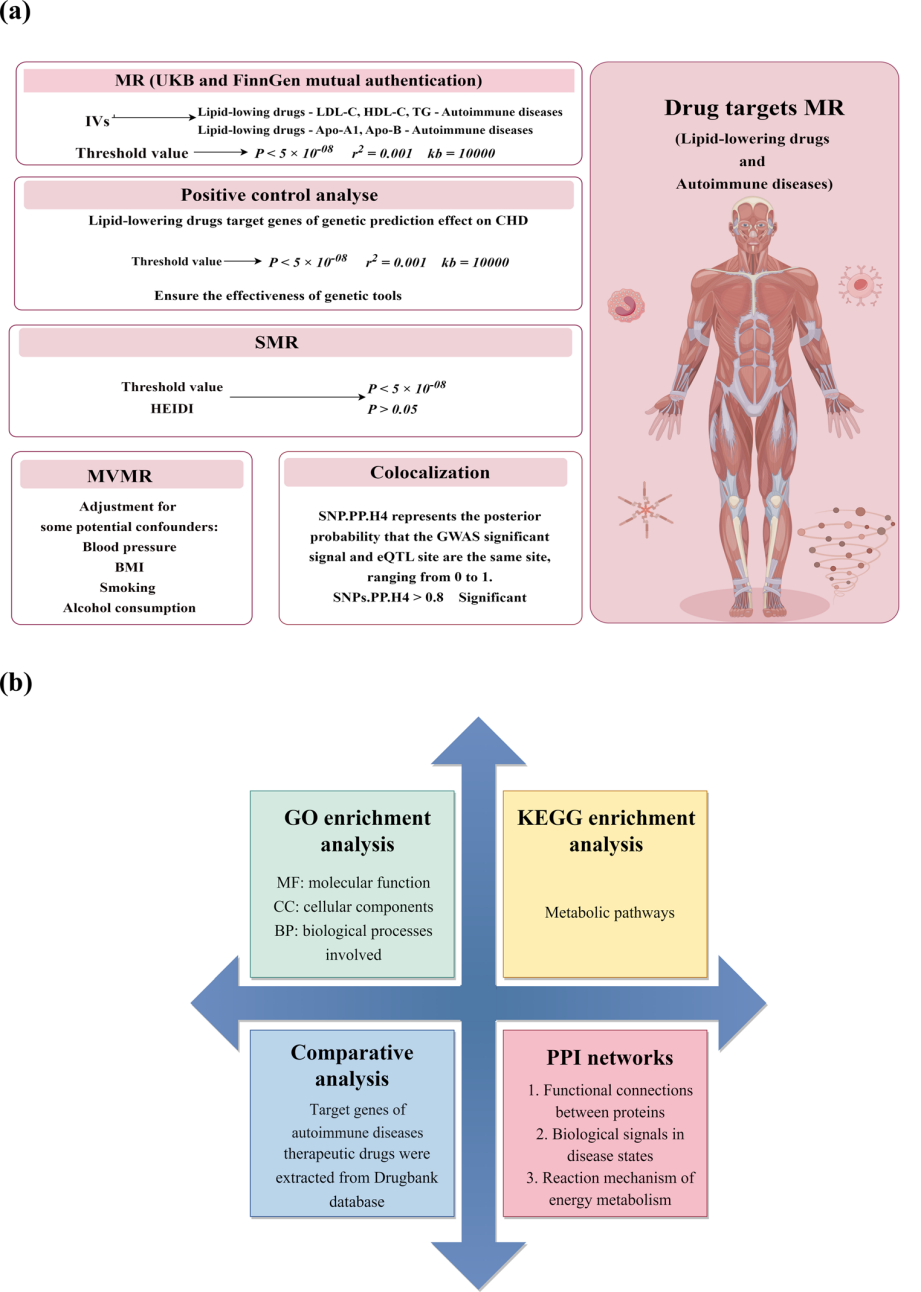
Overview of the study design (**a**) Identification of genetic associations using Mendelian randomization. (**b**) Constructions of gene enrichment and network analysis. ADs: Autoimmune diseases; Apo-A1: apolipoprotein A1; Apo-B: apolipoprotein B; BMI: body mass index; CHD: coronary heart disease; DBP: diastolic blood pressure; MR: Mendelian randomization; eQTL: Expression quantitative trait loci; IVs: instrumental variables; GO: Gene Ontology; KEGG: Kyoto Encyclopedia of Genes and Genomes; PPI: Protein-protein interaction; SMR: summary data-based MR; TSMR: two-sample MR; MVMR: multivariable MR; LDL-C: low-density lipoprotein cholesterol; TG: triglyceride (TG); HDL-C: high-density lipoprotein cholesterol; HEIDI: heterogeneity in dependent instruments

This study was designed following the Specifications for Reporting Observational Epidemiological Studies in MR (STROBE-MR) (Table S1) [19]. The data sources in the present study were derived from publicly available summary data of genome-wide association studies (GWAS) and eQTL data (Table S2). The study design is displayed in Fig. 1. Given that all datasets were freely available in the public domain, therefore, no additional ethical review was required.

### IVs selection

First, we identified and extracted independent genetic variants associated with low-density lipoprotein cholesterol (LDL-C), triglyceride (TG), and high-density lipoprotein cholesterol (HDL-C) in the largest GWAS from the Global Lipid Genetics Consortium (GLGC), according to the strict criteria (*P* < 5 × 10^− 08^, r^2^ < 0.001, kb = 10,000). To verify the reliability of the results, genome-wide associated genetic variants of additional two lipid traits, apolipoprotein A1 (Apo-A1) and apolipoprotein B (Apo-B), were selected from the database of MRC Integrative Epidemiology Unit (MRC-IEU) to construct a new set of IVs, where Apo-A1 is the crucial transporter protein associated with HDL-C, and Apo-B is the transporter protein associated with LDL-C and TG formation. The detailed information regarding the selected IVs for pharmacological genes was listed in Table S3-4. Second, to identify the lipid-lowering effects of the drug target, the genes encoding the pharmacological targets of lipid traits were comprehensively screened in Drug Bank database (https://go.drugbank.com/). A total of 12 drug targets genes relevant to lipid traits (LDL-C, TG, HDL-C, Apo-A1 and Apo-B) was captured, including the target genes for lowering LDL-C and Apo-B levels (HMGCR [3-hydroxy-3-methylglutaryl-CoA reductase], PCSK9, NPC1L1, ABCG5/ABCG8 [ATP binding cassette subfamily G member 5/8], LDLR [low-density lipoprotein receptor], APOB [apolipoprotein B]), target genes for lowering TG and Apo-B levels (LPL [lipoprotein receptor], ANGPTL3 [angiopoietin-like 3], PPARA [peroxisome proliferator-activated receptor alpha] and APOC3 [apolipoprotein C3]), and target genes for enhancing HDL-C and Apo-A1 levels (CETP [cholesterol ester transfer protein]) (Table S3). Moreover, we extracted single-nucleotide polymorphisms (SNPs) within a ± 100 kb window region of the gene location in lipid traits that were robustly associated with LDL-C, TG, HDL-C, Apo-A1 and Apo-B (*P* < 5 × 10^− 08^) using the GWAS data from GLGC and MRC-IEU, according to the methodology of the previous study (Tables S4) [20]. To enhance the statistical power and strength of the genetic instruments for each drug target, SNPs were then clumped to represent lipid-lowering drug targets if they were allowed to be in low linkage disequilibrium (LD) (R^2^ < 0.3) with a physical distance threshold of 250 kb and an *F*-statistic > 10 (Tables S5-6). Given that no eligible SNPs were obtained within 100 kb upstream and downstream of the *PPAPA*, we excluded it from subsequent analyses. Ultimately, the remaining 11 drug targets were incorporated in the present study, including *HMGCR, PCSK9, NPC1L1, ABCG5/ABCG8, APOB, LDLR, LPL, APOC3, ANGPTL3* and *CETP*.

In addition, we extracted GWAS data of coronary heart disease (CHD) as a positive control to validate the robustness of genetic variants as drug targets from the CARDIoGRAMplusC4D consortium. The detailed information of included data sources was summarized in Table S2.

As for the observed positive causality between drug targets and ADs, we used the summary-level eQTLs data in whole blood or tissues that were derived from eQTLGen Consortium (https://www.eqtlgen.org/) or GTEx Consortium V8 (https://gtexportal.org/) to further validate the differential expression of target genes. The cis-eQTL SNPs were recognized as genetic instruments located within 500 kb encoding the target gene and significantly affects the expression of the drug target gene (*P* < 5 × 10^− 08^, r^2^ < 0.1, MAF > 0.05).

### Outcome sources

Summary genetic association data among five types of ADs, including RA, SLE, multiple sclerosis (MS), ulcerative Colitis (UC), and Crohn’s disease (CD), were obtained from the MRC-IEU Open GWAS database (https://gwas.mrcieu.ac.uk/) at the University of Bristol and the FinnGen consortium. The sources of genetic data from the MRC-IEU database were defined as discovery datasets for the outcome, where the GWAS of RA included 2,5708 European individuals (5,539 cases and 20,169 controls) (ebi-a-GCST000679), the GWAS of SLE included 1,4267 European individuals (5,201 cases and 9,066 controls) (ebi-a-GCST003156), the GWAS of MS included 115,803 European individuals (47,269 cases and 68,374 controls) (ieu-b-18), the GWAS of UC included 26,405 European individuals (5,587 cases and 197,774 controls) (ebi-a-GCST000964), and the GWAS of CD included 51,874 European individuals (17,897 cases and 33,977 controls) (ieu-a-12) [21–25]. For replication analyses, independent summary data of ADs were derived from the FinnGen consortium (version 5), including seropositive RA (finn-b-RHEUMA_SEROPOS_STRICT), SLE (finn-b-M13_SLE), MS (finn-b-G6_MS), UC (finn-b-ULCERNAS), and CD (finn-b-K11_KELACROHN).

### Participant overlap assessment

During the causal inference of MR analysis, there has often been IV bias due to a high overlap between samples, which could lead to the possibility of a type 1 error [26]. The reliability and validity of MR analysis could be accepted with no or minor overlap between exposures and outcomes (sample overlap < 10%). In the present study, there was a lower sample overlap between the datasets of lipid traits/lipid-lowering drug targets and ADs (Supplementary Figure S1 & Supplementary Figure S2), indicating that the causal estimates were less likely to be affected by Winner’s curse bias.

### Statistical analyses

#### MR analysis

To ensure the validity of causal estimates, three fundamental assumptions should be fulfilled when performing MR analysis: (1) The selected IVs must be highly correlated with the exposure (the relevance assumption [27]. Here, the *F* statistic was used to evaluate the strength of the IVs–exposure correlation. The correlation with an *F* statistic > 10 was strong enough to avoid the weak IVs bias [28]; (2) The selected IVs should affect the outcome only through the exposure, not via other pathways (the exclusion restriction assumption) [29]. MR-Egger regression was used to identify the horizontal pleiotropy pathway between IVs and outcome [30]; (3) IVs should be independent of confounders (the independence assumption) [31].

The causal associations of lipid traits and lipid-lowering drug targets with the risk of ADs were rigorously assessed through a comprehensive univariable two-sample MR (TSMR) analysis with an inverse-variance weighted model, TSMR is a novel biostatistical approach that utilizes genetic variants from summary data across different populations to infer the causality between exposure factors and disease outcome. To validate the presence of expression-phenotype causal associations, additional SMR analysis was conducted to infer the causality between eQTL data of drug targets and ADs, which provides a more powerful estimate of the effect size of gene expression of genetic variants on the risk ADs. The magnitude of heterogeneity in the findings was tested using the heterogeneity in dependent instruments (HEIDI) tool (*P* > 0.05 was defined as the findings not being affected by heterogeneity) [32]. Considering the underlying influences of other risk factors on the causality between lipid traits/lipid-lowering drug targets and ADs, five common risk factors (systolic blood pressure [SBP], diastolic blood pressure [DBP], body mass index [BMI], smoking, and alcohol consumption) associated with ADs were identified from previous studies, and TSMR analyses were implemented to verify the causal associations between these factors and ADs; as for those risk factors that had a causal relationship with ADs, we then used MVMR analysis to evaluate the direct effect of lipid traits or lipid-lowering drug targets on ADs risk after controlling for the influence of these risk factors, where MVMR uses genetic variants as IVs to estimate the direct causal effects of multiple exposures on an outcome simultaneously [33].

Several sensitivity analyses were conducted to ensure the stability of the findings. First, the essential prerequisite for satisfying causal inference is the absence of horizontal pleiotropy, which was then tested using the MR Pleiotropy Residual Sum and Outlier (MR-PRESSO) method and results with a *P*-value greater than 0.05 were identified as the absence of horizontal pleiotropy [34]. Second, we used the Cochran *Q* statistic to rule out heterogeneity between IVs, and a *P*-value greater than 0.05 indicated no heterogeneity [35]. In addition, we performed leave-one-out cross-validation to rule out if any single SNPs significantly affect the stability of causal estimates [36].

Considering the multiple tests when inferring the causal associations of lipid traits or lipid-lowering drug targets with five types of ADs, Bonferroni correction was employed to avoid the possible false-positive results (type I errors) due to multiple tests. In the TSMR analysis for lipid traits and ADs, *P* < 0.002 (5 lipid traits, 5 ADs) was set as the presence of causal associations; as for the TSMR analysis between lipid-lowering drug targets and ADs, *P* < 0.001 (10 target genes, 5 ADs) was defined as the evidence of significant causality. In addition, as for other statistical analyses, *P* < 0.05 was considered to be statistically significant.

### Colocalization analysis

To resolve possible cascade imbalances and false-positive results, we performed co-localization analyses between 10 drug target genes and ADs to ensure that genetic variants only influence the phenotype by altering gene expression *via* the lipids pathway. Default a priori probabilities were P1 = 1 × 10^− 04^, P2 = 1 × 10^− 04,^ and P12 = 1 × 10^− 05^. A posteriori probability corresponding to one of the basic assumptions of co-localization, where H0 indicates the probability that neither feature in the region is genetically associated, and H1/H2 suggests the probability that either phenotype one or phenotype two are genetically related in that region, H3 shows the probability that both features are related but have different causal variables, and H4 represents the probability that both features share a causal variable. The co-localization analysis provides the magnitude of the probability that the two traits are affected by the same causal variant with the calculation of H4.

### Enrichment analysis

To investigate the biological relevance and functional characterization of potential drug targets, the Kyoto Encyclopedia of Genes and Genomes (KEGG) and gene ontology (GO) enrichment analyses were performed [37]. KEGG provides a systematic analysis of gene function and pathways in terms of genes and molecules, and GO offers a broader classification of the molecular function (MF), cellular components (CC), and biological processes involved (BP) in genes or gene products.

### Comparative analysis and protein interaction network construction

To comprehensively understand the possible interplays between lipid-lowering targets and the approved therapeutic drug targets of ADs, we summarized the information about the currently approved drug targets for ADs from Drugbank. The protein-protein interaction (PPI) networks were constructed to reveal the functional linkages between target gene coding proteins and lipid-lowering drug targets and to investigate biological signaling and energy metabolism response mechanisms involved in autoimmune response.

All aforementioned statistical analyses were performed using “TwoSampleMR”, “MVMR”, “COLOC” and “clusterProfiler” packages in R4.2.2. The SMR analyses were performed using the software tool developed by Yang Lab under the Linux environment. Comparative analysis was carried out based on an online gene search tool, STRING (https://string-db.org/), with a minimum confidence score of 0.4 and defaults for the remaining parameters. In addition, the BioGRID database was used for PPI construction (https://thebiogrid.org/), and Cytoscape (v3.9.1) was applied to visualize PPI results.

## Results

### Causal associations between lipid traits and ADs

A number of 80 SNPs associated with LDL-C, 56 SNPs associated with TG, 90 SNPs associated with HDL-C, 73 SNPs associated with Apo-A1, and 56 SNPs associated with Apo-B were recognized as candidate IVs for lipid traits (Table S4).

**Fig. 2:**
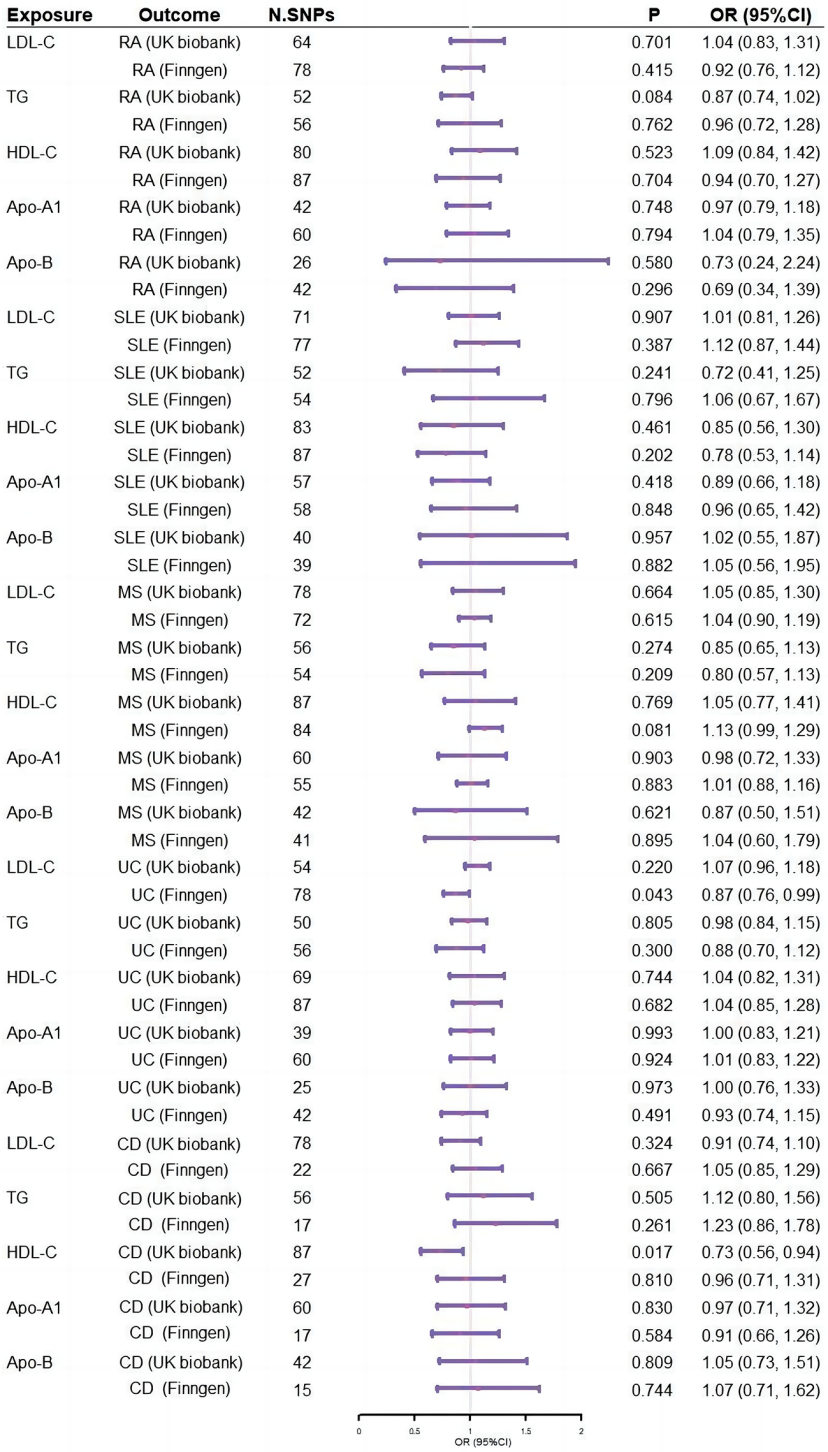
Forest plot of association of lipid traits with risk of ADs. ADs: Autoimmune diseases; RA: Rheumatoid arthritis; SLE: Systemic lupus erythematosus; MS: Multiple sclerosis; UC: ulcerative colitis; CD: Crohn’s disease; Apo-A1: apolipoprotein A1; Apo-B: apolipoprotein B; LDL-C: low-density lipoprotein cholesterol; TG: triglyceride (TG); HDL-C: high-density lipoprotein cholesterol; N.SNPs: number of single-nucleotide polymorphisms; OR: odds ratio

The results indicated that genetically determined HDL-C increase was causally associated with the risk of developing CD in discovery datasets (odds ratio [OR] = 0.73, 95%CI: 0.56, 0.94, *P* = 0.017) (Fig. 2 & Table S8), but this finding was not validated in replicate CD datasets. No causality between other lipid traits and risk of CD was observed (Fig. 2 & Table S8). In terms of the associations between lipid traits and other ADs, we did not observe any causal links of LDL-C, TG, HDL-C, Apo-A1 and Apo-B with the risks of RA, SLE, MS, and UC (Fig. 2 & Table S9-12). Heterogeneity tests revealed the presence of heterogeneity when inferring causal estimates of lipid traits and five types of ADs, the intercept term in MR-Egger regression supported that there was no overall horizontal pleiotropy (Table S13).

### Causal associations between lipid-lowering drug targets and ADs

A total of 11 lipid-lowering drug targets genes was identified, including *HMGCR, NPC1L1, PCSK5, ABCG5/ABCG8, APOB, LDLR, LPL, APOC3, ANGPTL3* and *CETP*, the strength and variance explanation of selected IVs proxies for each drug target were displayed in Tables S5-6. To ensure the effectiveness of IVs proxy to lipid-lowering drug targets, the correlations between drug targets related SNPs and CHD were validated as the positive control (Table S7). It showed that there were causal associations between 10 drug targets (HMGCR, NPC1L1, PCSK9, APOB, ABCG5/ABCG8, LDLR, APOC3, LPL and CETP) and CHD. Unfortunately, we did not observe a causal relationship between ANGPTL3 inhibition and CHD, thus, the target gene of ANGPTL3 was removed for further analysis (Table S7).

**Fig. 3:**
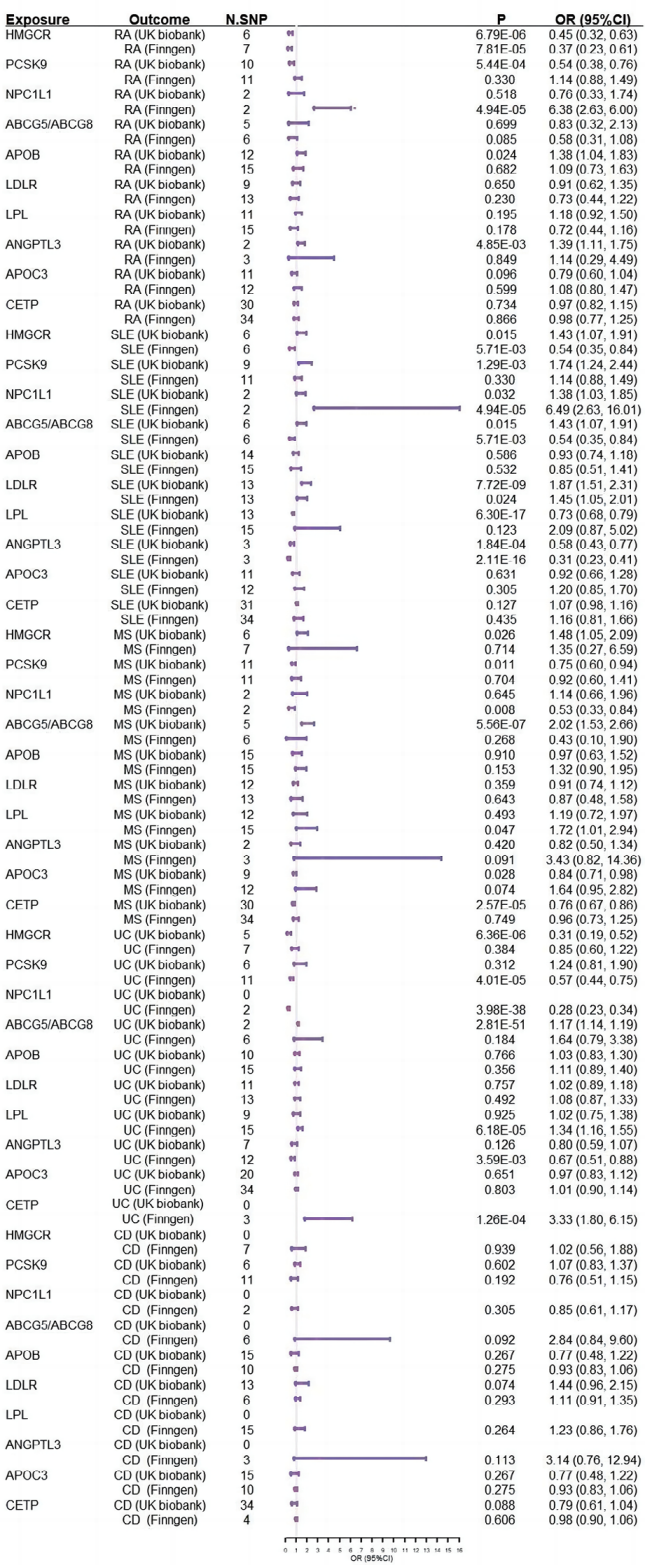
Forest plot of associations of genetically proxied drug targets with risk of ADs. HMGCR: 3-hydroxy-3-methylglutaryl-CoA reductase; PCSK9: proprotein convertase subtilisin/kexin type 9; NPC1L1: Niemann-Pick C1-like intracellular cholesterol transporter 1; ABCG5/ABCG8: ATP binding cassette subfamily G member 5/8; LDLR: low-density lipoprotein receptor; APOB: apolipoprotein B; LPL: lipoprotein receptor; ANGPTL3: angiopoietin-like 3; APOC3: apolipoprotein C3; ADs: Autoimmune diseases; RA: Rheumatoid arthritis; SLE: Systemic lupus erythematosus; MS: Multiple sclerosis; UC: ulcerative colitis; CD: Crohn’s disease; N.SNPs: number of single-nucleotide polymorphisms; OR: odds ratio

Genetically proxied HMGCR inhibition equivalent to per standard deviation (SD) decrease in LDL-C was causally associated with a lower risk of RA in both discovery (OR = 0.45, 95%CI: 0.32, 0.63, *P* = 6.79 × 10^− 06^) and replicate datasets (OR = 0.37, 95%CI: 0.23, 0.61, *P* = 7.81 × 10^− 05^) after Bonferroni correction (Fig. 3 & Table S14-15 & Supplementary Fig. 3). Nevertheless, we did not observe the causal associations of other genetic mimicry drug targets (*NPC1L1, PCSK5, ABCG5/ABCG8, APOB, LDLR, LPL, APOC3* and *CETP*) and RA, neither did the genetically proxied drug targets (*HMGCR, NPC1L1, PCSK5, ABCG5/ABCG8, APOB, LDLR, LPL, APOC3* and *CETP*) inhibition and SLE, MS, CD and UC (Table S16-23).

The results of MR Egger regression and MR-PRESSO showed no evidence of pleiotropy and underlying outliers (all *P* > 0.05), and sensitivity analyses with the leave-one-out method demonstrated that the causal estimates were stable after excluding any individual SNPs (Table S24).

### Gene expression and colocalization analysis

Given the protective effects of genetic variants in HMGCR on RA, the summary-level eQTL data in whole blood or multi-tissues were used to further validate the causality. The results of SMR analysis implied that genetically proxied HMGCR inhibition was associated with a lower risk of RA in whole blood (OR = 0.48, 95%CI: 0.29, 0.82, *P* = 6.86 × 10^− 03^, HEIDI = 0.125) and skeletal muscle site (OR = 0.75, 95%CI: 0.56, 0.99, *P* = 4.48 × 10^− 02^, HEIDI = 0.187) (Table S25).

Colocalization analyses were then performed to determine the likelihood whether genetic variants associated with HMGCR expression in relevant tissues shared causal loci with RA, and we found that for LDL and RA within the HMGCR gene, the respective probabilities of H4 were 22.22%; for Apo-B and RA within the HMGCR gene, the respective probabilities were 29.64% (Supplementary Fig. 4 & Table S26). These findings indicated that these two traits might not be affected by the same causal variant.

### MVMR analysis

Based on the observed causal association between HMGCR and RA, further MVMR was performed to evaluate the direct causal effect of HMGCR on RA after adjusting for the underlying risk factors. The selected common risk factors of blood pressure, BMI, smoking, and alcoholic drinks were validated whether there was the presence of causality with RA in using TSMR analysis, and results showed that SBP, BMI, and smoking were causally associated with RA (Table S27). Hence, these factors were incorporated into subsequent MVMR analysis, and the findings indicated that HMGCR suppression was causally linked to a lower RA risk after controlling for the influence of SBP, BMI, and smoking (OR = 0.33, 95%CI = 0.40, 0.96, *P* = 0.042) (Table S28).

### Enrichment analysis

**Fig. 4:**
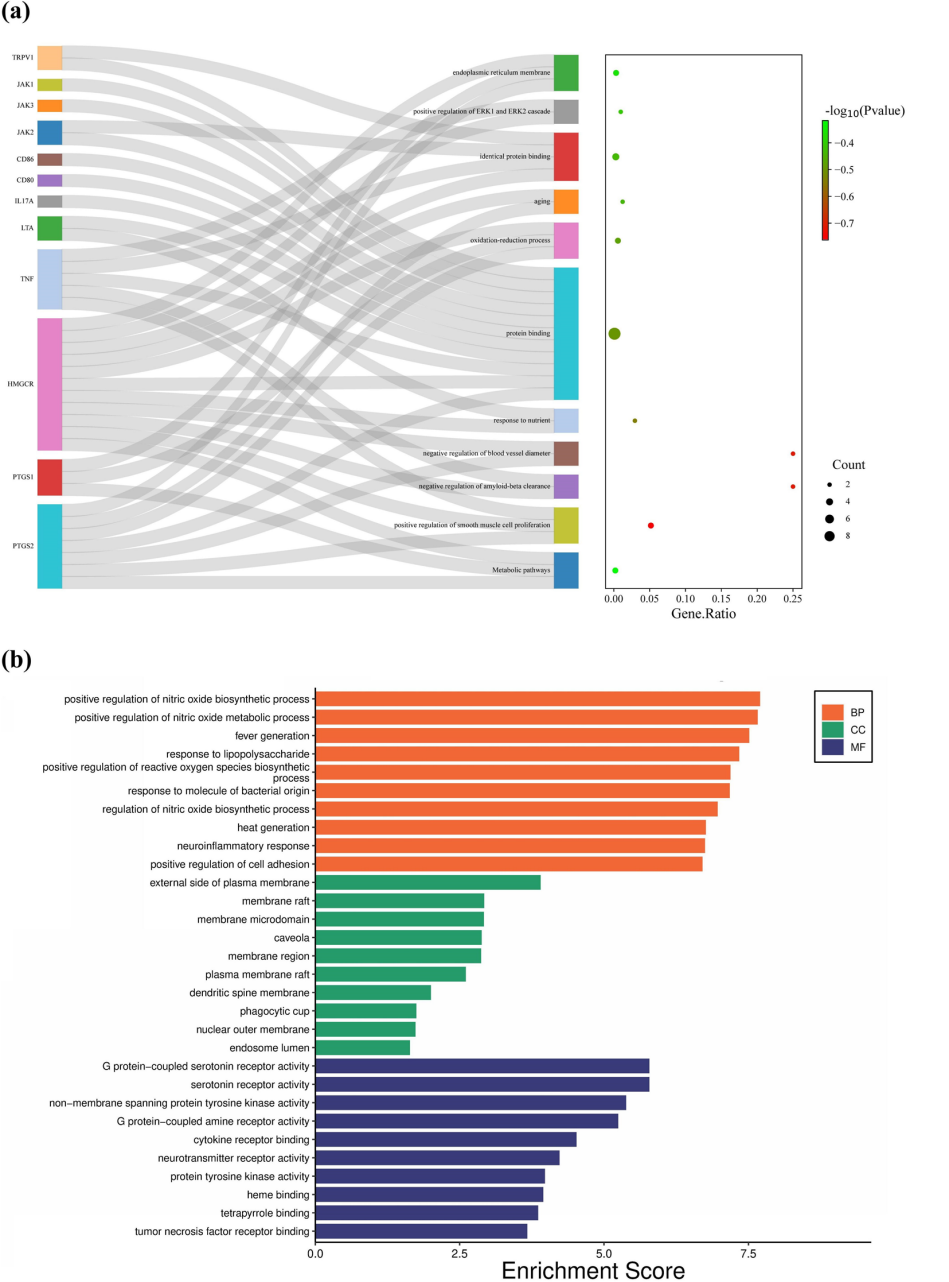
Functional interactions between HMGCR gene and the approved RA drug targets. (**a**) The combination diagram of Sankey and bubbles depicts the action mechanism of HMGCR; (**b**) GO enrichment results for three terms. HMGCR: 3-hydroxy-3-methylglutaryl-CoA reductase; MF: molecular function; CC: cellular components; BP: biological processes

To understand the possible interactions between HMGCR and the approved drug targets of RA, the gene function was evaluated. Go enrich analyses indicated that the function of these genes was closely associated with the regulation of nitric oxide biosynthesis and metabolism, response to lipopolysaccharide in BP, membrane raft and microdomain, caveola in CC, and serotonin receptor activity, G protein-coupled serotonin receptor activity and cytokine receptor binding in MF. KEGG pathway analysis showed that HMGCR and the approved drug targets of RA were mainly involved in the pathogenesis of type 1 diabetes (T1D), leishmaniasis, necroptosis, etc. (Fig. 4 & Supplementary Figs. 5–8 & Table S29-30).

### Comparative analysis and PPI networks

**Fig. 5:**
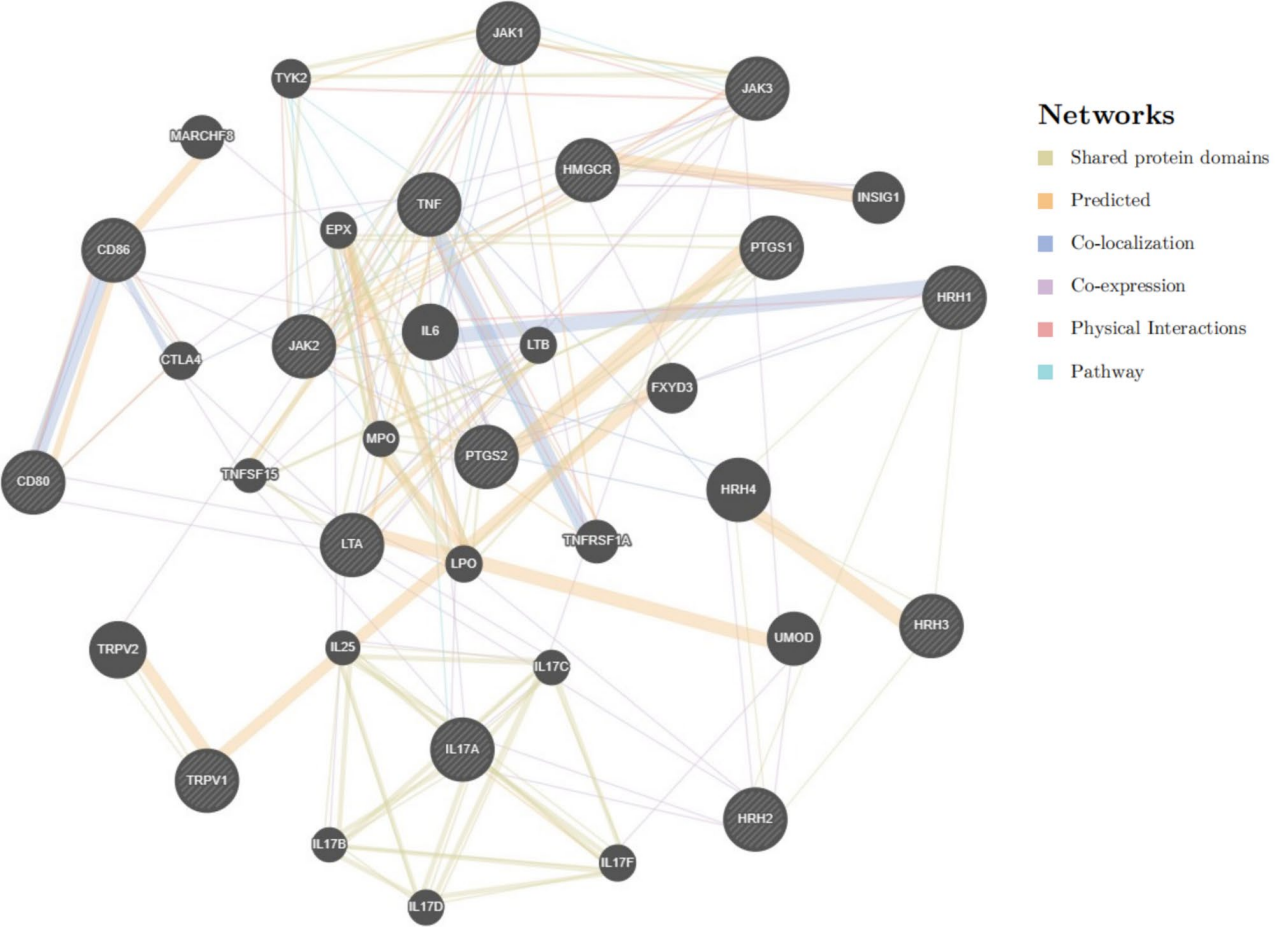
PPI network between HMGCR and the approved drug targets of RA. HMGCR: 3-hydroxy-3-methylglutaryl-CoA reductase; PPI: Protein–protein interaction; RA: rheumatoid arthritis

The PPI network was constructed with the use of HMGCR and the approved drug targets of RA, the results discovered 18 potentially interactive genes that were co-associated with each other. These linkages included co-expression (78.77%), common protein structural domains (25.90%), co-localization (19.70%), and physical interactions (13.34%). The results of the functional analysis of the PPI network were in line with the findings of enrichment analysis, suggesting the potential functional correlations of HMGCR with the molecular mechanism of RA pathogenesis (Fig. 5).

## Discussion

Recent evidence has highlighted the significant roles of lipid metabolism disorders in the development and progression of ADs [7, 38]. For instance, Zhang et al. reported that lipid-lowering therapy with simvastatin could reduce interleukin-17 A (IL-17 A) expression in CD4^+^ T cells and inhibit the activation of T cells towards to T helper 17 cells (Th17), suggesting the potential anti-inflammatory and immunomodulatory effects of statins [39]. Additionally, active RA patients exhibited rapid deterioration in lipid profiles, with decreased HDL-C levels and elevated atherogenic lipoproteins like Apo-B [40, 41]. It has been revealed that the majority of MS patients showed a disturbed systemic lipid metabolism, and the changes in total cholesterol (TC) or fatty acid might serve as biomarkers of disease activity and progression of this disease. Previous studies have demonstrated that SLE patients had a high risk of dyslipidemia, and an abnormal lipid metabolism was associated with the disease activity. The above evidence indicates that the dysfunction of lipid metabolism might play a significant role in the development and progression of ADs.

Our study was conducted to comprehensively understand the relationships of lipid traits and lipid-lowering drug targets with the risks of ADs with the implementation of various biostatistical approaches, including TSMR, SMR, and MVMR analyses. We did not observe causal associations between lipid traits and risks of ADs in both discovery and replicate datasets. Nevertheless, in terms of lipid-lowering drug targets, the genetically proxied HMGCR inhibition equivalent to LDL-C decrease was causally associated with a lower risk of RA, SMR analysis also supported the presence of an association between HMGCR gene suppression and RA. Furthermore, after adjusting for the underlying confounding factors, MVMR analysis demonstrated a direct causal effect of HMGCR inhibition on RA, suggesting that HMGCR-mediated LDL-C inhibition might play as a protective role in the lowering risk of RA. In addition, functional enrichment analyses implied that HMGCR was involved in the regulation of extracellular signal-regulated kinases 1/2 (ERK1/2) cascade, ageing, and oxidation-reduction processes, and PPI network analysis revealed that HMGCR was predominantly interlinked with Janus kinase 3 (JAK3) [already known drug targets for ADs] and insulin-inducible gene 1 (INSIG1) [key regulatory factor of lipid metabolism], suggesting that HMGCR might serve as a promising therapeutic target for RA.

**Fig. 6:**
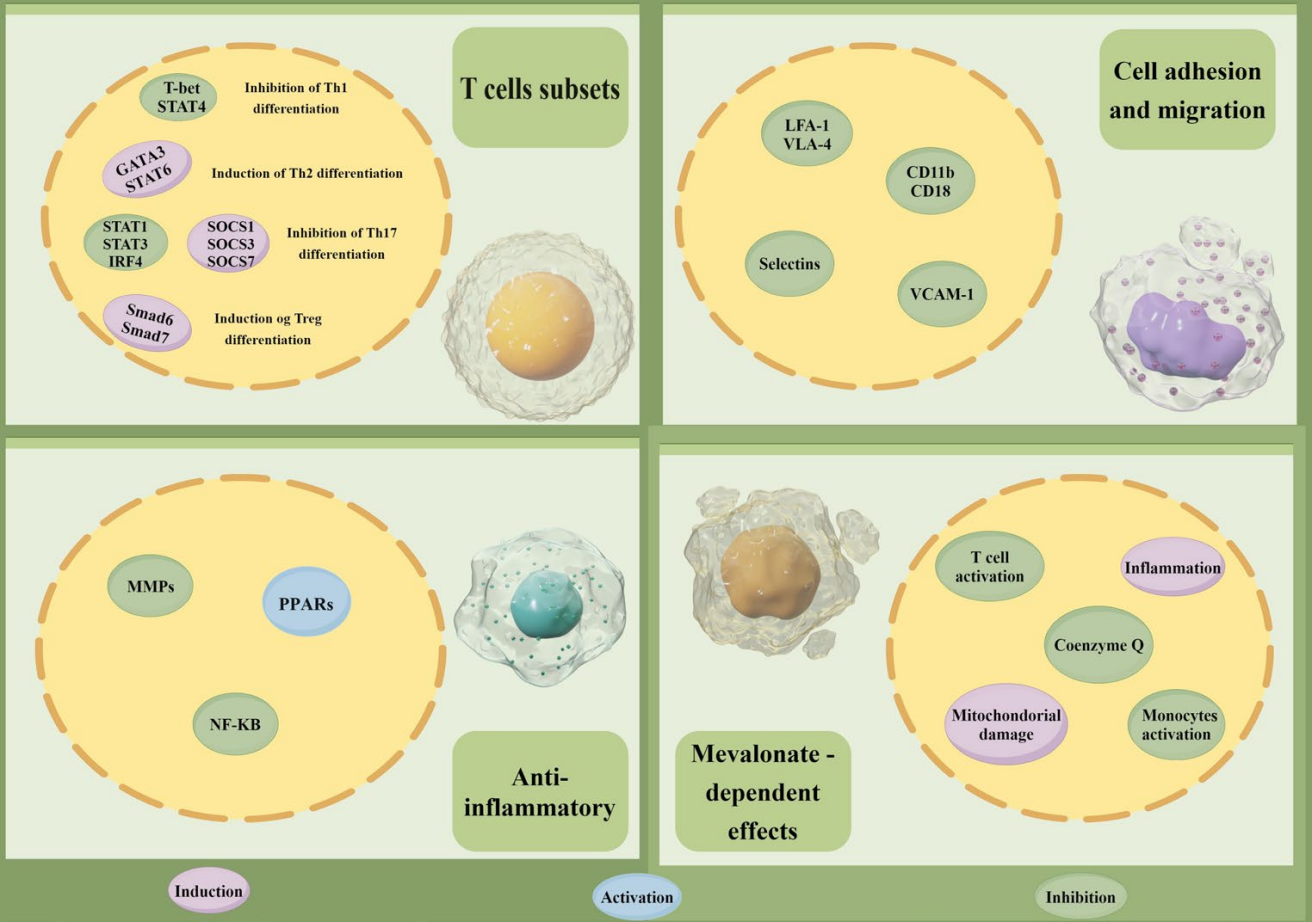
The immunoregulatory effects of statins. STAT4: signal transducer and activator of transcription 4; GATA3: GATA binding protein 3; STAT6: signal transducer and activator of transcription 6; STAT1: signal transducer and activator of transcription 1; STAT3: signal transducer and activator of transcription 3; IRF4: interferon regulatory factor 4; SOCS1: suppressor of cytokine signaling 1; SOCS3: suppressor of cytokine signaling 3; SOCS7: suppressor of cytokine signaling 7; Smad6: Smad Family Member 6; Smad7: Smad Family Member 7; MMPs: matrix metalloproteinases; PPARs: peroxisome proliferator-activated receptors; NF-κB: nuclear transcription factor-kappa B; LFA-1: lymphocyte function-associated antigen 1; VLA-4: very late appearing antigen-4; CD11b: CD11 antigen-like family member b; CD18: integrin β2 subunit; VCAM1: vascular cell adhesion molecule 1

HMGCR is the rate-limiting enzyme in the cholesterol biosynthesis pathway, the inhibitors of HMGCR exert a therapeutic effect on lowering LDL-C by suppressing HMG-CoA reductase activity, and are commonly used for the treatment of CVD in clinical practice [42]. In recent years, emerging evidence has shown the anti-inflammatory properties beyond the cholesterol-lowering effects of HMGCR inhibitors in the treatment of ADs. Statins, as one of the classic HMGCR inhibitors, are not only used to lower cholesterol levels in the blood but also show an anti-inflammatory effect. Established studies have revealed that statins can affect immune cell trafficking (reducing adhesion of immune cells to the endothelium), apoptosis (inducing programmed cell death in immune cells), and differentiation (modulating the T cells differentiation) through inhibiting the activation of nuclear factor-kappa B (NF-кB) and the subsequent production of pro-inflammatory factors, such as tumor necrosis factor-alpha (TNF-α), interleukin-6 (IL-6) and interleukin-1 beta (IL-1β) (Fig. 6) [43, 44]. Numerous studies have explored the use of statins in the treatment of RA. Although some studies have indicated potential benefits, such as disease remission and an improvement of joint inflammation, the findings have been inconsistent, and several studies did not report significant improvements. A recent systemic review provided a clinical dosing profile of statins in ADs, suggesting promising therapeutic benefits of lipid-lowering treatment in various ADs, especially in RA and SLE [45]. Moreover, considering an increased CVD risk in RA patients, it has been suggested that statins, with dual effects in lipid-lowering and immune-modulating, may be a potential priority drug for reducing CVD risk in patients with RA [46, 47].

In our study, we observed that HMGCR inhibition was correlated with a lower RA risk, this might be explained by the following aspects. First, HMGCR plays a pivotal role in the synthesis of cholesterol and fatty acids, which are essential components of cell membranes and signaling molecules. The inhibition of HMGCR with the use of statins could lead to alterations in the metabolism of these lipids, potentially affecting cellular signaling pathways associated with cell growth, survival, and apoptosis, such as the PI3K/Akt and MAPK/ERK pathways [48, 49]. Second, the alterations in lipid metabolism, due to HMGCR inhibition, could also modulate inflammatory and immune responses by reducing the recruitment and activation of various immune cells, including T cells, B cells and macrophages [50]. In addition, HMGCR is also involved in the regulation of oxidative stress and the antioxidant defense system. HMGCR inhibition could disrupt the balance between oxidative stress and antioxidant defense, potentially reducing the oxidative stress that contributes to RA pathogenesis [51]. Statins, commonly known as HMGCR inhibitors, have shown an anti-inflammatory effect beyond lipid-lowering properties in RA patients. It has been revealed that treatment with simvastatin could reduce several serum inflammatory markers in RA patients [52]. This finding was also observed in a recent meta-analysis, where Ren et al. showed that the use of statins can reduce inflammatory markers of C-reactive protein (CRP) and erythrocyte sedimentation rate (ESR), as well as alleviate the disease activity and symptoms of RA patients [53]. Taken together, considering the anti-inflammatory and lipid-lowering properties, statins might be used as a supplement to existing RA treatment regimens, and provide additional benefits in controlling disease activity and improving patient outcomes.

Currently, emerging evidence has highlighted that biologics are cornerstones of the treatment of RA, such as TNF-α and IL-6 receptor inhibitors [47]. There are, however, as compared to conventional disease-modifying anti-rheumatic drugs (DMARDs) and non-steroidal anti-inflammatory drugs (NSAIDs), the higher annual costs and opportunistic infections of biologic therapy might pose significant barriers to patient access to necessary treatments. Targeting HMGCR with lipid-lowering drugs represents a promising therapeutic approach for RA as compared with biologics. These classic drugs offer the potential for novel applications in RA treatment, as they are cost-effective and widely accessible [54].

There were still several limitations that should be noticed. First, our study was conducted based on the European population due to data availability, while MR offers a robust method for estimating causal effects, the selection of specific ancestry of the populations should be taken into account to ensure the generalizability of the findings. Second, genetic variation reflects the long-term effects of changes in lipid levels on the risk of ADs, which may not directly translate into the short-term efficacy of lipid-lowering drugs. MR only provides insights into causal associations and their direction rather than making quantitative estimates. Third, as the association between target gene expression and risk of ADs was conducted using cis-eQTL data, it should be acknowledged that ADs are influenced by a complex interplay of multiple factors, and the exclusive focus on cis-eQTL associations may not capture the full spectrum of biological influences. Furthermore, the enrichment analyses rely on predefined sets of valuable genes that may not explain whole possible biological mechanisms underlying the causes of ADs. It is essential to notice that the colocalization analysis showed a relatively low probability that HMGCR-mediated lipid traits and RA share the same causal variant, suggesting that the observed association between HMGCR lipid-lowering targets and RA may not be directly driven by a shared genetic basis.

## Conclusions

Our study reveals that HMGCR-mediated LDL-C lowering and HMGCR expression inhibition h*ave* causal relationships with a lower risk of RA, but none of the lipid traits and lipid-lowering targets were found to causally associate with the risk of SLE, MS, UC, and CD. The findings of our study suggest that HMGCR might be a promising therapeutic target for RA, and the early modulation of LDL-C may mediate its biological mechanism to achieve both lipid-lowering and immune-modulating effects in RA. However, further validation across ethnic and molecular mechanism exploration is still necessary to unveil the therapeutic potential of HMGCR for RA.

### Electronic supplementary material

Below is the link to the electronic supplementary material.

Supplementary Material 1

Supplementary Material 2

## Data Availability

The data and material that support the findings of this study are available from public datasets that could be found in NHGRIEBI GWAS Catalog and Yang Lab.

## Abbreviations

ADs: Autoimmune diseases
Apo-A1: Apolipoprotein A1
Apo-B: Apolipoprotein B
BMI: Body mass index
CHD: Coronary heart disease
CRP: C-reactive protein
DBP: Diastolic blood pressure
MR: Mendelian randomization
eQTL: Expression quantitative trait loci
ESR: Erythrocyte sedimentation rate
IVs: Instrumental variables
GO: Gene Ontology
KEGG: Kyoto Encyclopedia of Genes and Genomes
PPI: Protein-protein interaction
SMR: Summary data-based MR
TSMR: Two-sample MR
MVMR: Multivariable MR
LDL-C: Low-density lipoprotein cholesterol
TG: Triglyceride
HDL-C: High-density lipoprotein cholesterol
HEIDI: Heterogeneity in dependent instruments
SBP: Systolic blood pressure
STROBE-MR: Specifications for Reporting Observational Epidemiological Studies in MR
GWAS: Genome-wide association studies
GLGC: Global Lipid Genetics Consortium
MRC-IEU: MRC Integrative Epidemiology Unit
RA: Rheumatoid arthritis
SLE: Systemic lupus erythematosus
MS: Multiple sclerosis
UC: Ulcerative colitis
CD: Crohn’s disease
HMGCR: 3-hydroxy-3-methylglutaryl-CoA reductase
PCSK9: Proprotein convertase subtilisin/kexin type 9
NPC1L1: Niemann-Pick C1-like intracellular cholesterol transporter 1
ABCG5/ABCG8: ATP binding cassette subfamily G member 5/8
LDLR: Low-density lipoprotein receptor
APOB: Apolipoprotein B
LPL: Lipoprotein receptor
ANGPTL3: Angiopoietin-like 3
APOC3: Apolipoprotein C3
SNPs: Single-nucleotide polymorphisms
IL-17A: Interleukin-17 A
Th17: T helper 17 cells
ERK1/2: Extracellular signal-regulated kinases ½
JAK3: Janus kinase 3
INSIG1: Insulin-inducible gene 1
NF-кB: Nuclear factor-kappa B
TNF-α: Tumor necrosis factor-alpha
IL-6: Interleukin-6; IL-1β:interleukin-1 beta
DMARDs: Disease-modifying anti-rheumatic drugs
NSAIDs: Non-steroidal anti-inflammatory drugs
STAT4: Signal transducer and activator of transcription 4
GATA3: GATA binding protein 3
STAT6: Signal transducer and activator of transcription 6
STAT1: Signal transducer and activator of transcription 1
STAT3: Signal transducer and activator of transcription 3
IRF4: Interferon regulatory factor 4
SOCS1: Suppressor of cytokine signaling 1
SOCS3: Suppressor of cytokine signaling 3
SOCS7: Suppressor of cytokine signaling 7
Smad6: Smad Family Member 6
Smad7: Smad Family Member 7
MMPs: Matrix metalloproteinases
PPARs: Peroxisome proliferator-activated receptors
LFA-1: Lymphocyte function-associated antigen 1
VLA-4: Very late appearing antigen-4
CD11b: CD11 antigen-like family member b
CD18: Integrin β2 subunit
VCAM1: Vascular cell adhesion molecule 1
