# Supplementary material for: Identification of lipid-modifying drug targets for autoimmune diseases: insights from drug target mendelian randomization
Source: Lipids Health Dis. 2024 Jun 22;23:193. doi: 10.1186/s12944-024-02181-2 (PMC11193261; doi:10.1186/s12944-024-02181-2)

**Supplementary Fig. 1 The sample overlap between lipid-lowering drug targets and ADs.**

(a) The sample overlap between exposures (lipid-lowering drug targets from GLGC) and outcomes (ADs from UK biobank); (b) The sample overlap between exposures (lipid-lowering drug targets from GLGC) and outcomes (ADs from Finngen); (c) The sample overlap between exposures (lipid-lowering drug targets from MRC-IEU) and outcomes (ADs from UK biobank); (d) The sample overlap between exposures (lipid-lowering drug targets from MRC-IEU) and outcomes (ADs from Finngen).

ADs: Autoimmune diseases; RA: Rheumatoid arthritis; SLE: Systemic lupus erythematosus; MS: Multiple sclerosis; UC: ulcerative colitis; CD: Crohn’s disease; MRC-IEU: MRC Integrative Epidemiology Unit; GLGC: Global Lipid Genetics Consortium


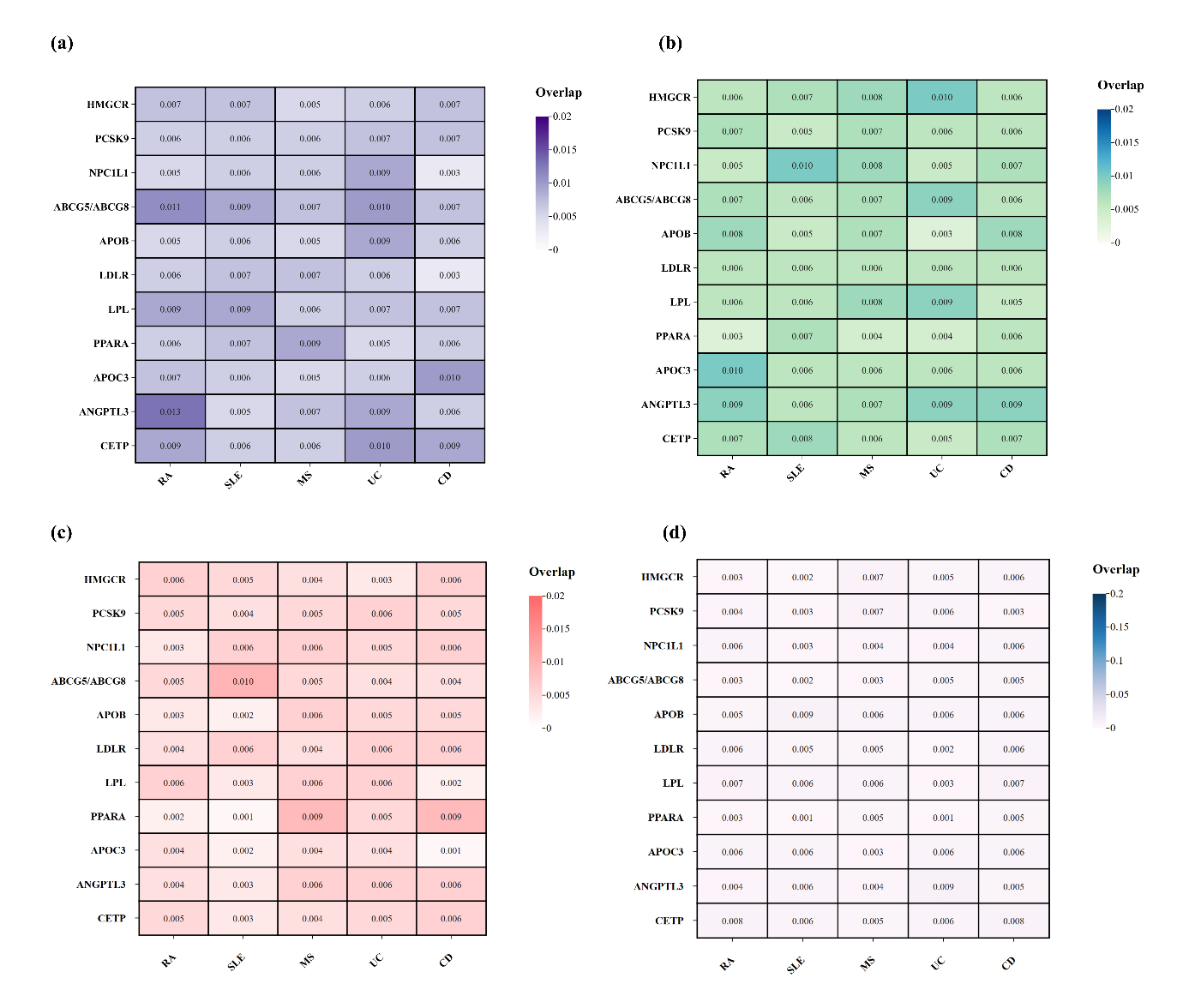


**Supplementary Fig. 2 The sample overlap between lipid traits and ADs.**

(a) The sample overlap between exposures (LDL-C, TG, HDL-C) and outcomes (ADs from UK biobank); (b) The sample overlap between exposures (LDL-C, TG, HDL-C) and outcomes (ADs from Finngen); (c) The sample overlap between exposures (Apo-A1, Apo-B) and outcomes (ADs from UK biobank); (d) The sample overlap between exposures (Apo-A1, Apo-B) and outcomes (ADs from Finngen).

ADs: Autoimmune diseases; RA: Rheumatoid arthritis; SLE: Systemic lupus erythematosus; MS: Multiple sclerosis; UC: ulcerative colitis; CD: Crohn’s disease; Apo-A1: apolipoprotein A1; Apo-B: apolipoprotein B; LDL-C: low-density lipoprotein cholesterol; TG: triglyceride (TG); HDL-C: high-density lipoprotein cholesterol


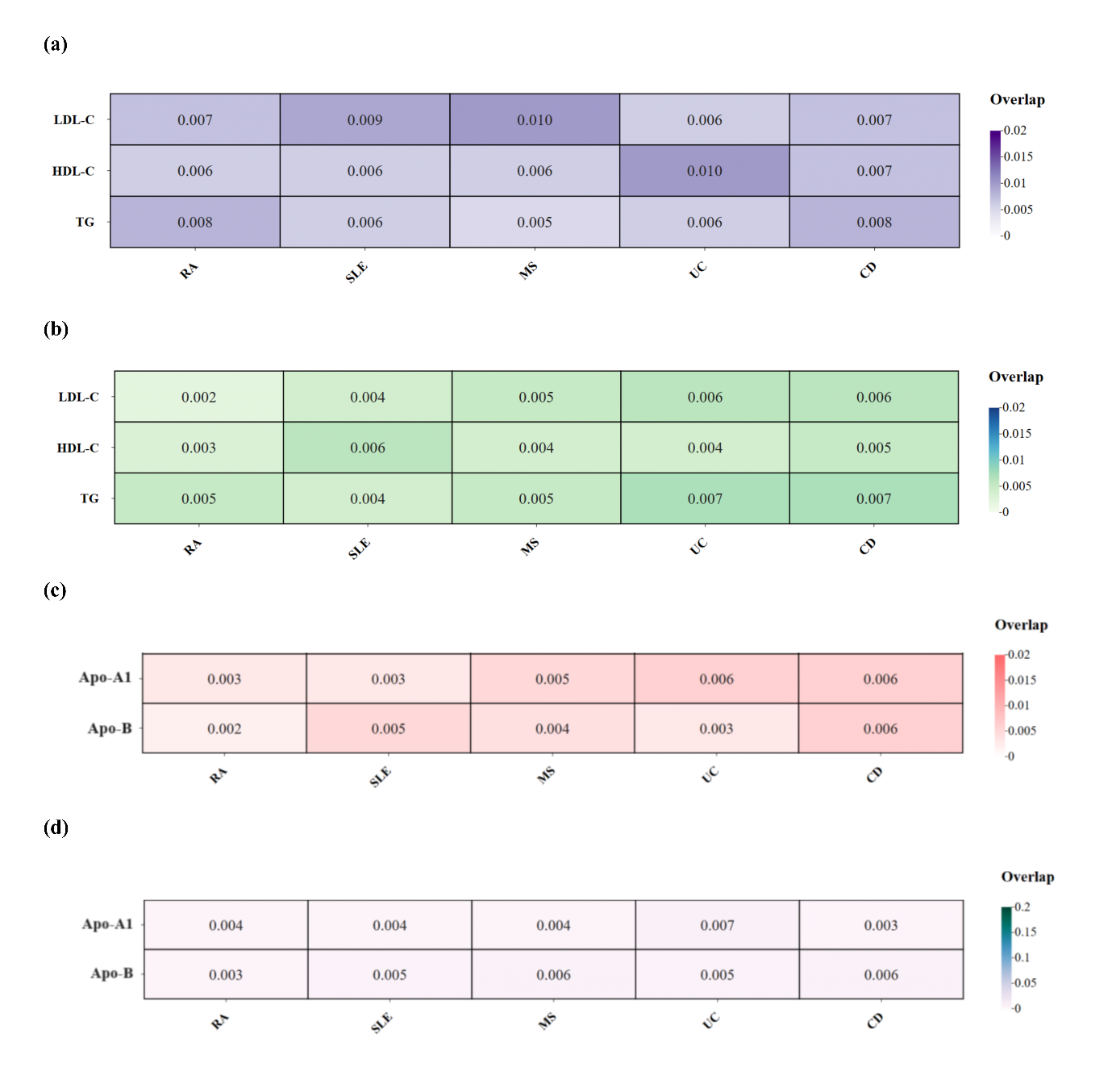


**Supplementary Fig. 3 SMR results for probe-centred genomic regions**

SMR: summary data-based Mendelian randomization


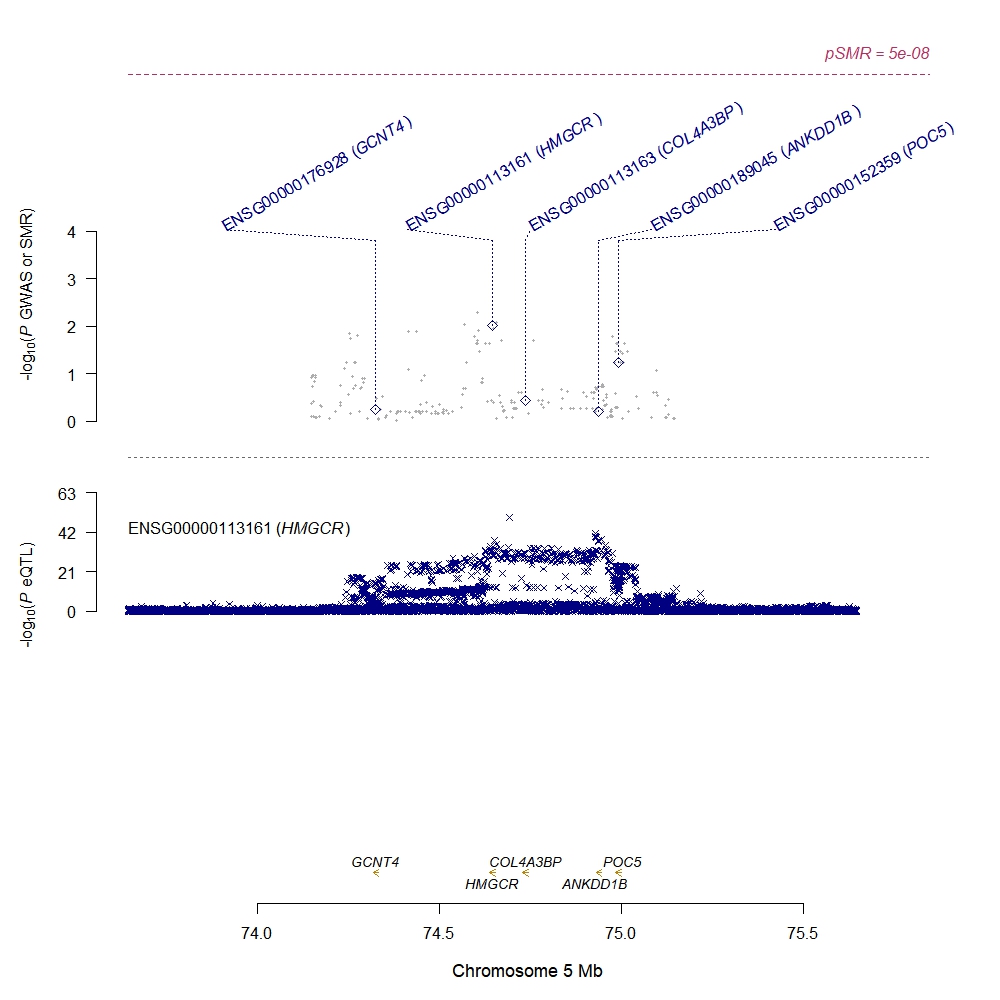


**Supplementary Fig. 4 Colocalization analysis between HMGCR-mediated lipid traits and RA**

(a) Colocalization analysis between HMGCR-mediated LDL-C and RA; (b) Colocalization analysis between HMGCR-mediated Apo-B and RA

HMGCR: 3-hydroxy-3-methylglutaryl-CoA reductase; LDL-C: low-density lipoprotein cholesterol; Apo-B: apolipoprotein B; RA: Rheumatoid arthritis


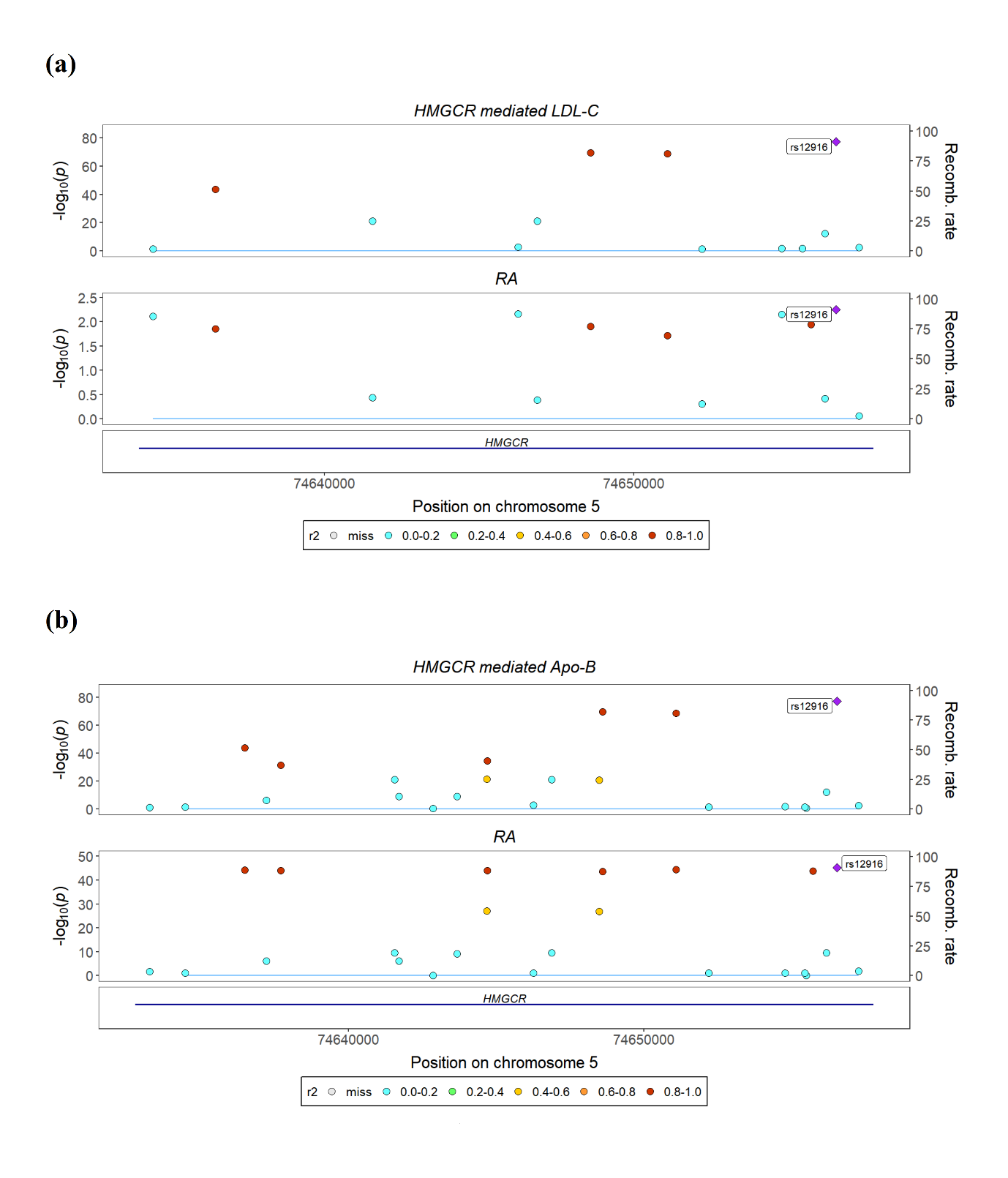


**Supplementary Fig. 5 Bubble plots of GO domains in cellular components**

GO: Gene Ontology


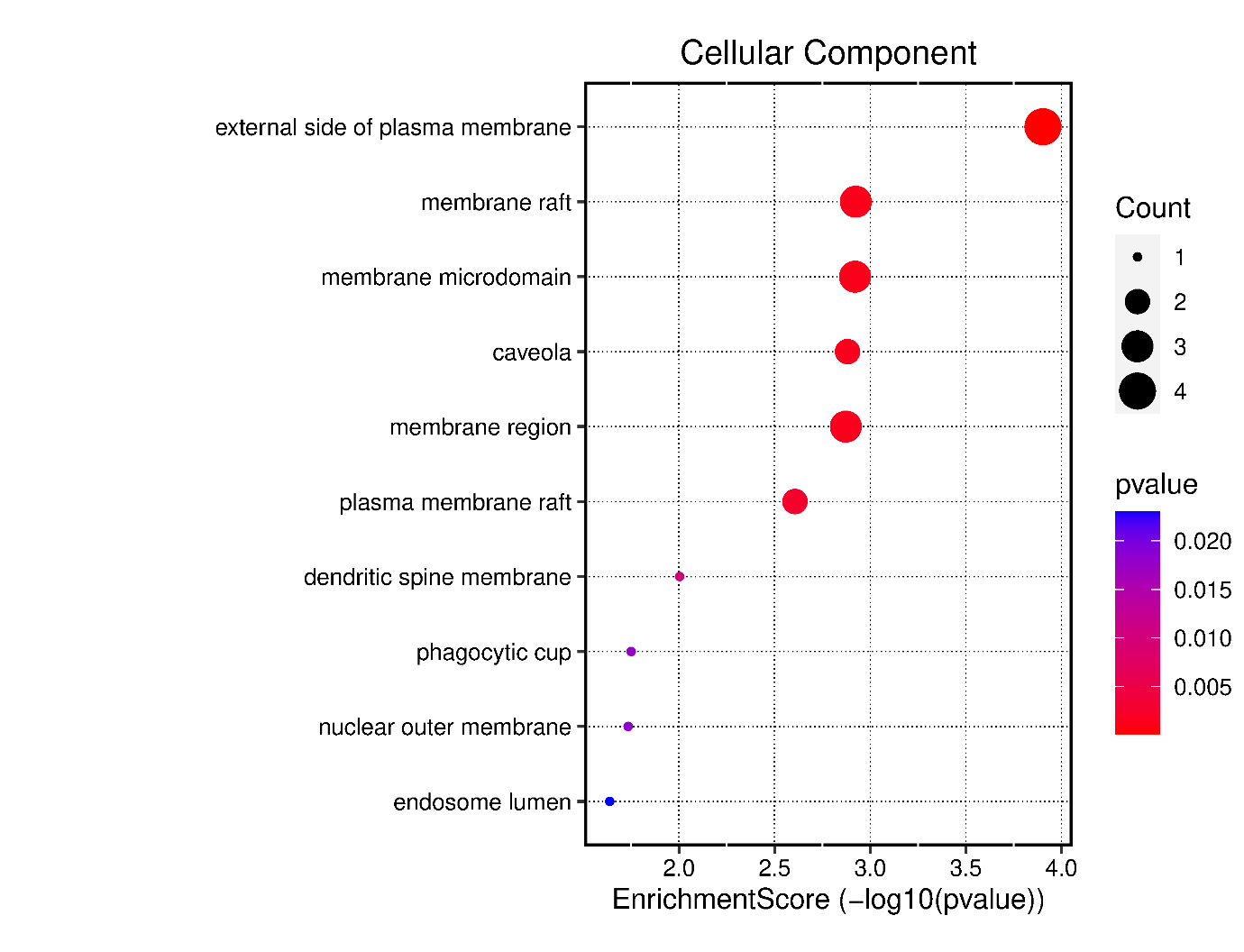


**Supplementary Fig. 6 Bubble plots of GO domains in biological processes**

GO: Gene Ontology


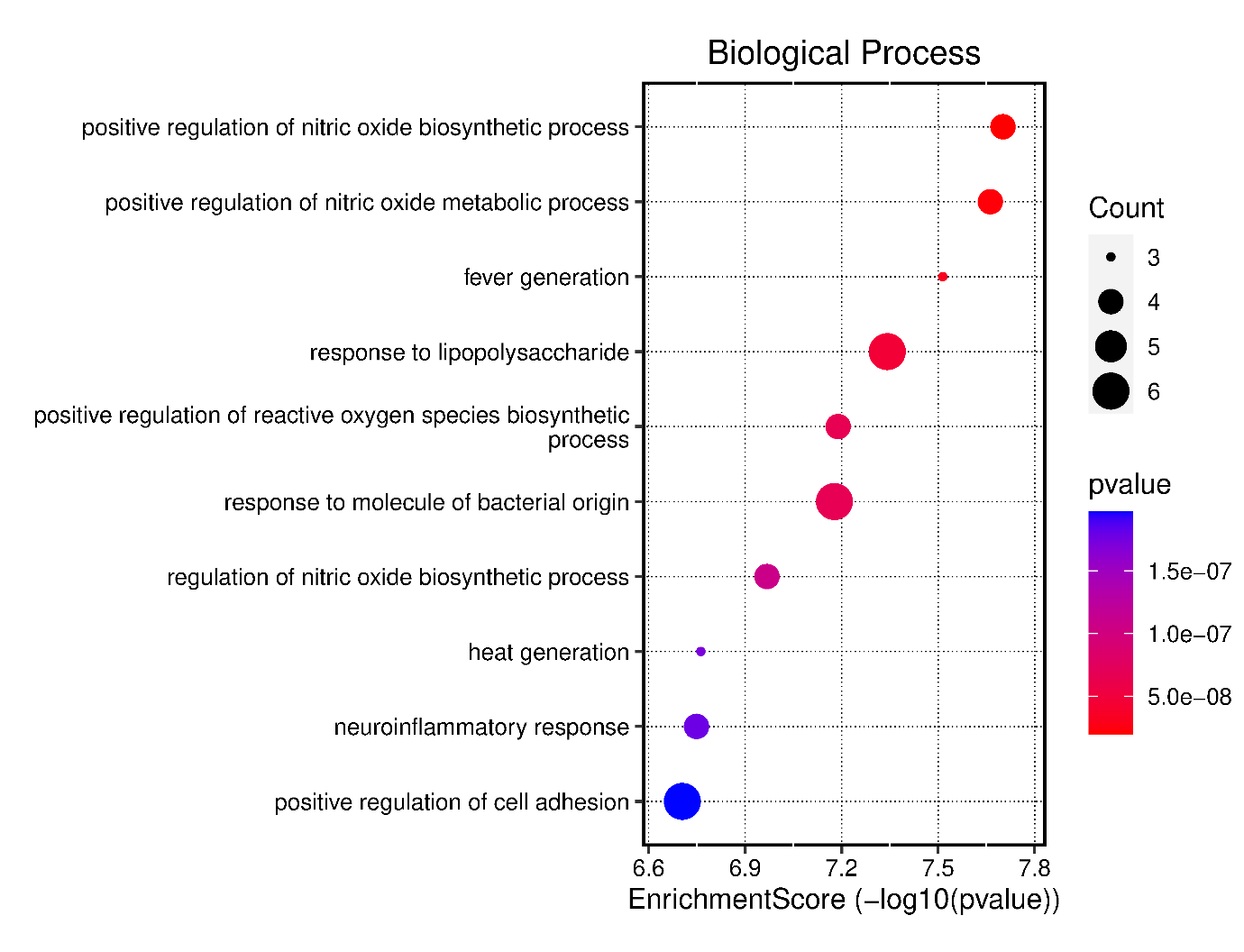


**Supplementary Fig. 7 Bubble plots of GO domains in molecular function**

GO: Gene Ontology


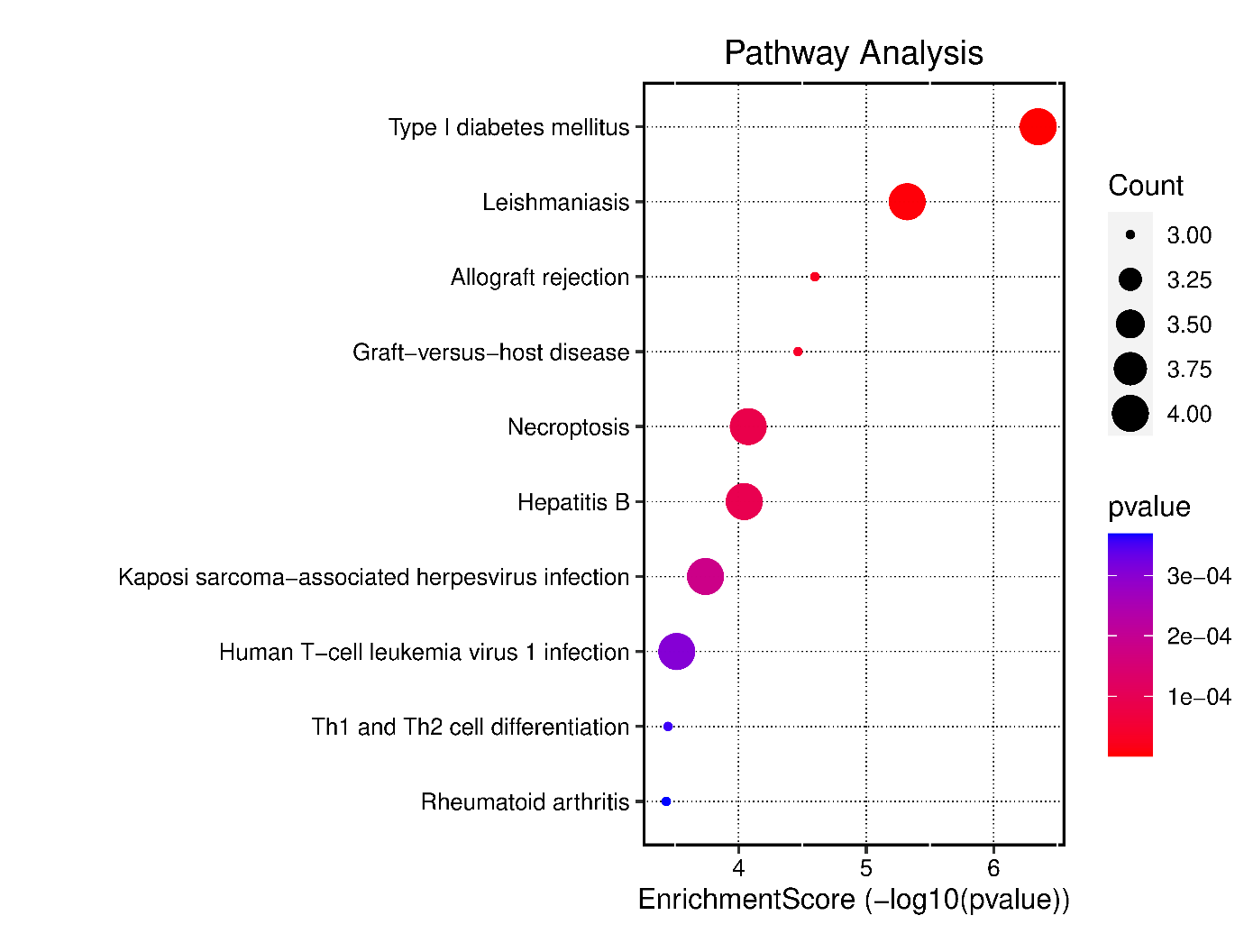


**Supplementary Fig. 8 Bubble plots of KEGG pathway analysis**

KEGG: Kyoto Encyclopedia of Genes and Genomes


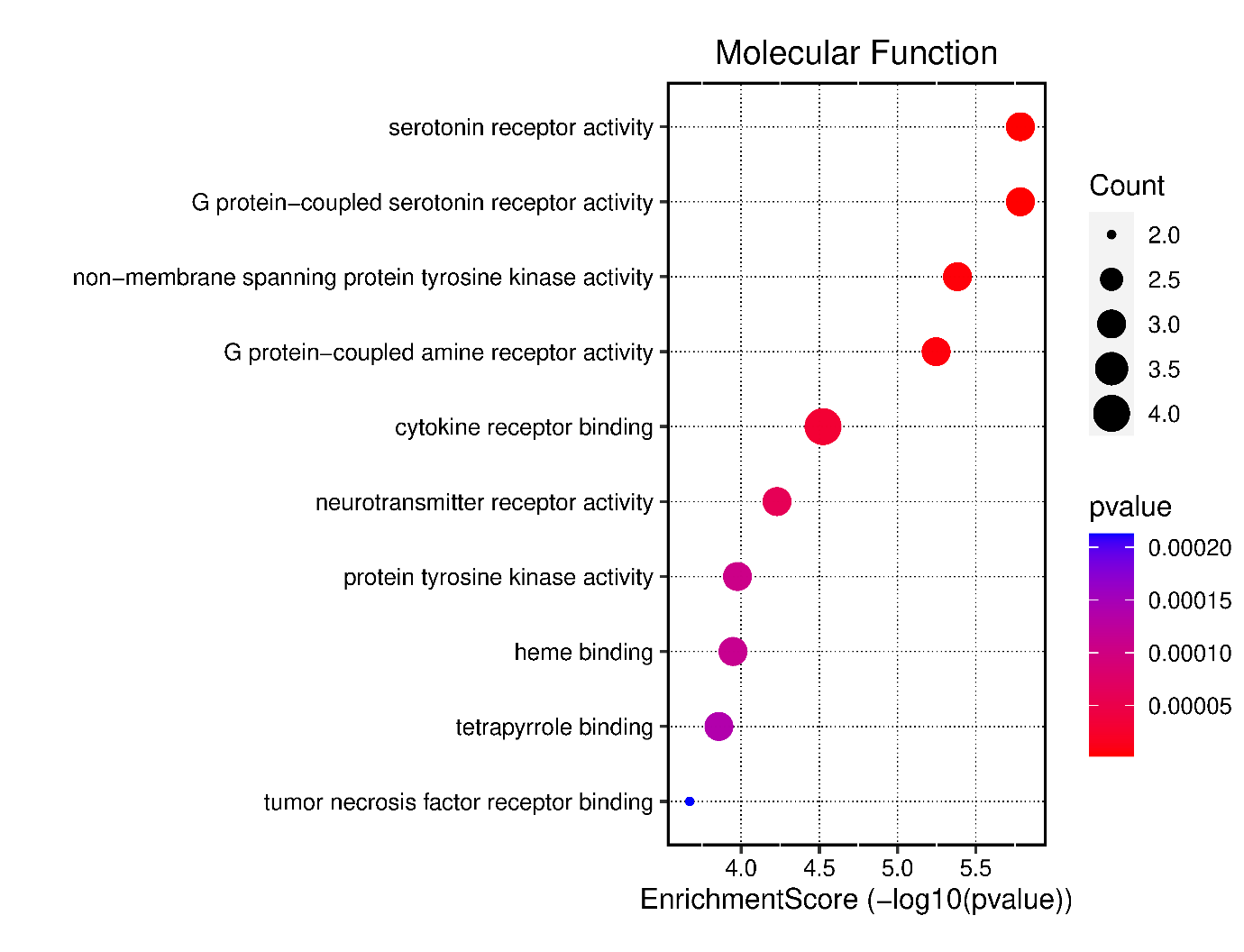

Supplement: Supplementary file 1 — Supplementary Material 1 [file 12944_2024_2181_MOESM1_ESM.docx]
